# 421. *In vitro* and *in vivo* synergistic antimicrobial activities of the combinations of meropenem, colistin, tigecycline, and ceftolozane/tazobactam against carbapenem-resistant *Klebsiella pneumoniae*

**DOI:** 10.1093/ofid/ofac492.497

**Published:** 2022-12-15

**Authors:** Young Kyung Yoon, Jeong Yeon Kim, Jin Woong Suh, Jang Wook Sohn

**Affiliations:** Korea University College of Medicine, Seoul, Seoul-t'ukpyolsi, Republic of Korea; Korea University College of Medicine, Seoul, Seoul-t'ukpyolsi, Republic of Korea; Korea University College of Medicine, Seoul, Seoul-t'ukpyolsi, Republic of Korea; Korea University College of Medicine, Seoul, Seoul-t'ukpyolsi, Republic of Korea

## Abstract

**Background:**

The aim of this study was to evaluate the *in vitro* and *in vivo* activities of various antimicrobial combinations against carbapenem-resistant *Klebsiella pneumoniae* (CRKP).

**Methods:**

The *in vitro* activity of six two-drug combinations against CRKP isolates collected from the rectal swab samples of patients with intestinal CRKP colonization was evaluated using the checkerboard method and time-kill assay to identify potential synergistic and bactericidal two-drug combinations against CRKP isolates. The *in vivo* efficacy of the combinations of colistin, meropenem and ceftolozane/tazobactam (C/T) as therapeutic options, was assessed in a mouse model of intraperitoneal CRKP sepsis. Antimicrobial susceptibility and genotypes of carbapenem-resistance were also analyzed.

**Results:**

A total of 5 clinical isolates of CRKP were collected from nonduplicate patients with CRKP infection. Of 5 CRKP isolates, 4 carried the *bla*_KPC-2_-type carbapenem-hydrolyzing enzymes except 1 *bla*_NDM-1_-type. Antimicrobial susceptibility rates were tigecycline 80%, colistin 40%, and C/T 0%, respectively. The checkerboard assay showed that *in vitro* synergistic activity against CRKP isolates was 90% for the meropenem-tigecycline combinations and 20% for colistin-C/T combinations. None of the various combinations of the six antibiotics showed a synergistic effect in the time-kill assay. However, the meropenem-colistin (100%) and colistin-C/T (100%) combinations showed bactericidal effects in the time-kill assay (using an antibiotic concentration of 1× MIC) (Table). The survival rates of the mice was 80% (4/5) at 48 h in the treated group using low dose combinations of colistin (10 mg/kg) and C/T (25 mg/kg), unlike both antibiotics, which were 60% in a monotherapy with the same dose (Figure).

Comparison of checkerboard assay and time-kill assay

**Figure:**
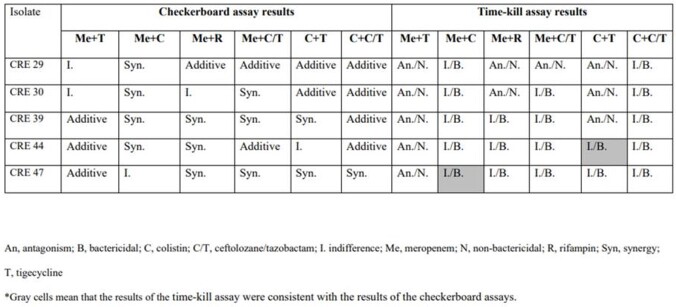

Comparison of checkerboard assay and time-kill assay associated with the bactericidal activity of two-drug combinations (1 × MIC) against each carbapenem-resistant Klebsiella pneumoniae isolate

In vivo survival tests for antimicrobial combinations

**Figure:**
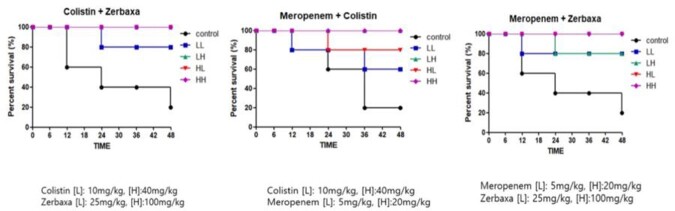

Comparison of the survival rates of the mice in the treated group using antimicrobial combinations

**Conclusion:**

In conclusion, the present *in vivo* study demonstrated that the low-dose combination of colistin and C/T may be a promising alternative to nephrotoxic high-dose colistin alone for treating CRKP infections. However, it was difficult to predict this possibility in *in vitro* tests, and even there is great discordance of antimicrobial synergistic activities between the checkerboard microdilution and time-kill assays.

**Disclosures:**

**All Authors**: No reported disclosures.

